# Post-traumatic stress disorder in Italy: a comprehensive evaluation of all the ICD comorbidities and gender-related differences

**DOI:** 10.1186/s13104-019-4792-0

**Published:** 2019-11-15

**Authors:** Fabio Ferretti, Andrea Pozza, Letizia Bossini, Serena Desantis, Miriam Olivola, Laura Del Matto, Giacomo Gualtieri, Roberto Gusinu, Daiana Bezzini, Andrea Fagiolini, Anna Coluccia

**Affiliations:** 1 Department of Medical Sciences, Surgery and Neurosciences, University of Siena, Viale Bracci 16, 53100 Siena, Italy; 2 Department of Molecular Medicine, University of Siena School of Medicine, Siena, Italy; 3 Department of Mental Health, University of Siena Medical Center (AOUS), Siena, Italy; 4 Health Service Management Board, Santa Maria alle Scotte University Hospital of Siena, Siena, Italy; 5 Department of Life Sciences, University of Siena, Siena, Italy

**Keywords:** Post-traumatic stress disorder, Comorbidity, Medical conditions, Gender differences, Diseases of the circulatory system, Metabolic syndrome, Neoplasms, ICD

## Abstract

**Objectives:**

The association between post-traumatic stress disorder (PTSD) and medical comorbidities is controversial since most studies focused on specific comorbidity and victim types. In Italy, data on this issue are scarce. A comprehensive evaluation of all the ICD medical categories co-occurring in PTSD may orient assessment and treatment during clinical and forensic practice. This is the first study evaluating all the ICD physical comorbidities and gender-related differences in Italian PTSD patients. Eighty-four PTSD patients (36 females, 48 males) were included. The Clinician-Administered PTSD Scale, Mini International Neuropsychiatric Interview and Davidson Trauma Scale were administered.

**Results:**

Most patients had a PTSD consequent to an accident and half of them presented extreme symptom severity. No gender differences emerged on symptom severity/duration and age at the event. Metabolic (39.29%), circulatory (20.24%) and musculoskeletal systems/connective tissue diseases (17.86%) were the most frequent comorbidities. Metabolic/circulatory diseases were more frequent among males (*p *= 0.019 and *p *= 0.027, respectively) while females more frequently showed neoplasms (*p *= 0.039). Physical comorbidities represent a serious complication in PTSD patients and are more prevalent than in the Italian population. While gender is not associated with symptom presentation, it seems to play a key role in specific comorbidities including metabolic, circulatory and neoplastic diseases.

## Introduction

Although it has been largely investigated in the literature, the association between post-traumatic stress disorder (PTSD) and medical comorbidities is still controversial, mainly because the majority of the studies focused on specific medical conditions [1–4] or populations such as veterans [5, 6]. According to a review [7], PTSD was associated with greater frequency and severity of cardio-respiratory and gastrointestinal complaints, while a recent study highlighted that chronic or neck problems, frequent headaches, arthritis or rheumatism and hypertension were highly prevalent in adults with PTSD [8]. A comprehensive investigation of all the physical conditions based on the International Classification of Diseases (ICD) may support assessment and treatment during clinical and forensic medical practice. In Italy, very few studies were dedicated to this topic, most of them related to psychological response to natural [9, 10] or human-made disasters [11] such as terroristic attacks. This is the first study assessing ICD physical comorbidities and the related gender differences in a group of Italian PTSD patients. This work extends the results of studies previously published by the authors [12, 13].

## Main text

### Methods

#### Study design and subjects

One-hundred eight subjects were assessed for inclusion from 2003 to 2017 at the Department of Psychiatry of Siena (*n *= 35) and at the Italian National Observatory for Victims of Terrorism (*n* = 73). Twenty-four subjects were excluded due to exclusion criteria. Thus, a sample of 84 subjects (average age = 50.36 ± 12.18, range = 19–78; 36 females and 48 males) was included. All subjects were diagnosed with PTSD according to DSM-IV criteria [14]. The following exclusion criteria were used: (a) comorbidity with any other psychiatric disorders, (b) intellectual disability, (c) neurological conditions, (d) being on concurrent psychotropic medication or psychotherapeutic treatment, (e) past treatment for PTSD (both medication and psychotherapy). The typologies of events faced by the subjects were classified according to the following categories [15]: war events, physical/sexual violence, accidents, unexpected death of a loved one, network event.

#### Measures

Socio-demographic and clinical data were collected by psychiatrists, together with a clinical interview for an overall analysis of the subject’s medical conditions. Diseases were classified according to the codes of The International Classification of Diseases, Ninth Revision, Clinical Modification (ICD9CM). The rationale for using ICD9CM categories was that this classification system was the most widely used one in clinical practice in Italy when the study started. The Clinician-Administered PTSD Scale [CAPS; [16] was administered by trained psychiatrists to confirm the diagnosis of PTSD, under the supervision of senior psychiatrists with 5-year experience in interviewing patients. Patients’ assessment was completed through the administration of other measures, whose results are not shown in this paper: the Mini International Neuropsychiatric Interview [MINI; [17] was used to assess comorbidity with other psychiatric disorders and the Davidson Trauma Scale [DTS; [18] was chosen to assess frequency and severity of post-traumatic symptoms.

#### Statistical analysis

Descriptive statistics were used to analyse patients’ characteristics based on gender. Fisher’s exact test explored the association of dichotomous categorical variables (positive/negative with regard to comorbidities) with gender, while Student’s *t*-test (two-tailed) and Mann–Whitney’s *U*-test (two-tailed) were chosen, according to the violation of normality assumption, to compare means of quantitative measures (CAPS total and subscale scores, PTSD duration and age at the event). For each ICD9CM category, the percentage of positive patients, and its 95% confidence interval, was computed, with a focus on the Metabolic syndrome, belonging to the Endocrine, nutritional and metabolic diseases. Although these percentages should not be considered a punctual estimation of prevalence since the study design was not cross-sectional, and neither a period estimation of prevalence, because of the wide period of patients’ recruitment, they have been considered as an approximation of the prevalence estimation. The significance level was set at *p *< 0.05 and the IBM-SPSS v23 was used for data analysis.

### Results

For the majority of the patients (Table 1), PTSD was diagnosed as a consequence of an accident (75.00% for females and 89.57% for males), a category including natural disasters and terrorist attacks. Severity of PTSD symptoms was similar across gender and half of the patients showed an extreme level of symptom severity (50.00% for females and 45.84% for males). The CAPS total score was slightly lower in males (76.69 ± 23.11) than in females (80.44 ± 30.42), although this difference was not significant. No significant gender differences were also found on PTSD symptoms, age at the event and duration of the disorder, even though males showed a longer duration (167.00 ± 171.19 months) than females (140.67 ± 146.21).

**Table 1:** gender comparison of patients’ characteristics: event type, PTSD severity categories, CAPS total score, CAPS subscale score, PTSD duration and age at the event

	Females (*n *= 36)	Males (*n *= 48)	Statistic	*p*
Event type^a^: *n* (%)				
War events	0 (0.00)	2 (4.17)		
Physical violence	0 (0.00)	1 (2.08)		
Sexual violence	1 (2.77)	1 (2.08)		
Accidents	27 (75,00)	43 (89,57)		
Unexpected death of loved one	6 (16,67)	1 (2.08)		
Network events	2 (5,56)	0 (0.00)		
CAPS categories^a^: n (%)				
Slight or sub-threshold	4 (11.11)	4 (8.33)		
Moderate	6 (16.67)	4 (8.33)		
Severe	8 (22.22)	18 (37.50)		
Extreme	18 (50.00)	22 (45.84)		
CAPS total score (mean ± SD)	80.44 ± 30.42	76.69 ± 23.11	*t*_(63)_ = 0.560	0.538
CAPS symptoms (mean ± SD)				
Re‐experiencing	23.89 ± 9.71	22.83 ± 7.56	*t*_(82)_ = 0.560	0.560
Avoidance and numbing	31.30 ± 14.38	30.00 ± 10.71	*t*_(62)_ = 0.458	0.649
Hyperarousal	25.25 ± 8.64	24.00 ± 8.04	*t*_(82)_ = 0.683	0.497
Duration of PTSD (months; mean ± SD)	140.67 ± 146.21	167.00 ± 171.19	MW U = 783.000	0.463
Age at the event (mean ± SD)	37.00 ± 13.64	37.60 ± 11.69	*t*_(82)_ = − 0.218	0.828

CAPS, Clinician-Administered PTSD Scale; MW U, Mann–Whitney U test; PTSD, Post Traumatic Stress Disorder

^a^No statistic was used to test the hypothesis of independence between categories (Chi squared), because more than 20% of the expected counts are less than 5 and not all individual expected counts are 1 or greater [19]

Diseases of endocrine, nutritional and metabolic systems showed a high frequency: 57.14% (CI 46.56–67.72) of the patients suffered from health problems related to this ICD9CM category, and 17.86% (CI 9.67–26.05) presented a Metabolic syndrome (Table 2).

**Table 2:** percentage of patients presenting comorbidities and gender comparison related to each disease

	% (All subjects) (95% CI)		Females (*n *= 36) *n* (%)	Males (*n *= 48) *n* (%)	p*
Metabolic syndrome (ICD Endocrine, nutritional and metabolic diseases)	17.86 (9.67 to 26.05)	Yes	2 (5.56)	13 (27.08)	0.019
		No	34 (94.44)	35 (72.92)	
ICD Diseases of the blood and blood‐forming organs and certain disorders involving the immune mechanism	10.71 (4.10 to 17.32)	Yes	5 (13.89)	4 (8.33)	0.488
		No	31 (86.11)	44 (91.67)	
ICD Diseases of the circulatory system	20.24 (11.65 to 28.83)	Yes	3 (8.33)	14 (29.16)	0.027
		No	33 (91.67)	34 (70.83)	
ICD Diseases of the skin and subcutaneous tissue	8.33 (2.42 to 14.24)	Yes	1 (2.78)	6 (12.50)	0.230
		No	35 (97.22)	42 (87.50)	
ICD Diseases of the digestive system	39.29 (22.29 to 42.29)	Yes	10 (27.78)	23 (47.92)	0.074
		No	26 (72.22)	25 (52.08)	
ICD Injury, poisoning and certain other consequences of external causes	2.38 (− 0.88 to 5.64)	Yes	0 (0.00)	2 (4.17)	0.504
		No	36 (100.00)	46 (95.83)	
ICD Certain infectious and parasitic diseases	3.57 (− 0.40 to 7.54)	Yes	0 (0.00)	3 (6.25)	0.256
		No	36 (100.00)	45 (93.75)	
ICD Diseases of the genitourinary system	13.10 (5.88 to 20.32)	Yes	5 (13.89)	6 (12.50)	1.000
		No	31 (86.11)	42 (87.50)	
ICD Endocrine, nutritional and metabolic diseases	57.14 (46.56 to 67.72)	Yes	17 (47.22)	31 (64.58)	0.125
		No	19 (52.78)	17 (35.42)	
ICD Neoplasms	8.33 (2.42 to 14.24)	Yes	6 (16.67)	1 (2.08)	0.039
		No	30 (83.33)	47 (97.92)	
ICD Diseases of the respiratory system	7.14 (1.63 to 12.65)	Yes	3 (8.33)	3 (6.25)	1.000
		No	33 (91.67)	45 (93.75)	
ICD Diseases of the nervous system	14.29 (6.81 to 21.77)	Yes	3 (8.33)	9 (18.75)	0.220
		No	33 (91.67)	39 (81.25)	
ICD Diseases of the musculoskeletal system and connective tissue	17.86 (9.67 to 26.05)	Yes	3 (8.30)	12 (25.00)	0.082
		No	33 (91.70)	36 (75.00)	
ICD Diseases of the eye and adnexa	2.38 (− 0.88 to 5.64)	Yes	1 (2.78)	1 (2.08)	1.000
		No	35 (97.22)	47 (97.92)	

* Fisher’s exact test (two-tailed)

Diseases of the digestive system (39.29%; CI 22.29–42.29), diseases of the circulatory system (20.24%; CI 11.65–28.83) and diseases of the musculoskeletal system and connective tissue (17.86%; CI 9.67–26.05) showed a high frequency as well. The ICD9CM categories with the lowest frequency rate were eye diseases and adnexa (2.38%) and certain infectious and parasitic diseases (3.57%), without considering those health problems due to external causes (e.g., injury, poisoning). Only three out of the fourteen diseases categories were significantly associated with gender. Diseases of the circulatory system were significantly more frequent in males (29.16%) than females (8.33%) (*p *= 0.027). Another significant association emerged for neoplasms but for this ICD9CM category, females showed a higher proportion (16.67%) than males (2.08%) (*p *= 0.039). Although the relationship between gender and the ICD code “Endocrine, nutritional and metabolic diseases” was not significant, the subcategory related to metabolic syndrome showed a significantly higher proportion of positive males (27.08%) than females (5.56%) (*p *= 0.019). None of the 95% confidence intervals computed by gender for these categories showed overlapping bounds.

### Discussion

Although in the literature there is a large number of studies concerning the association between PTSD and physical comorbidities, epidemiological data are scarce, particularly in Italy. In addition, previous investigations focused on specific types of comorbidities and there is a lack of a comprehensive assessment of all the ICD categories. The present study is the first one investigating all the ICD medical comorbid conditions and the related gender differences in Italian PTSD patients. Findings showed that specific comorbidities represent a serious issue in PTSD with metabolic, circulatory and musculoskeletal systems/connective tissue diseases being the most frequent ones. While gender is not associated with symptom presentation, it seems to play a key role in specific comorbidities, with males presenting a higher frequency of metabolic/circulatory diseases and females showing more frequently neoplastic conditions.

Comparisons with previous studies related to the prevalence of comorbidities in PTSD patients are difficult due to the different way of coding the pathologies: many studies focused on specific diseases (e.g., diabetes [20]) instead of all the ICD9CM categories that were used in this paper. Furthermore, existing findings about comorbidities prevalence showed a large degree of heterogeneity, partly due to the differences in study populations (e.g., veterans or general population). As mentioned before, in this study the percentage of positive patients was used as an approximation of the prevalence and this matter makes any comparison more inaccurate. Keeping in mind all these recommendations for the interpretation of the findings, the Italian group of PTSD patients showed interesting similarities and differences with other studies. In this group of subjects, findings showed a percentage of 17.86% of patients with metabolic syndrome, a proportion that was comparable to the study of Ching-En et al. [21] in Taiwan, where 12.91% of the PTSD population showed a comorbidity with this disease. Other studies reported much higher values of prevalence: according to the review of Rosenbaum and colleagues [22], the estimation of the metabolic syndrome prevalence in PTSD patients was 38.7% (CI 32.1–45.6%) and another study on Bosnian post-war PTSD showed a value of 48.3% [23].

The development of metabolic syndrome may be due to the stress of the trauma which acts through the neuropeptide Y and glucocorticoid systems [24]. Alternatively, it may be hypothesized that PTSD patients develop metabolic problems as a consequence of dysfunctional strategies used to cope with stress such as alcohol drinking [25].

Such a heterogeneity concerns other health-related problems as well. For example, diseases of the circulatory system were reported by 20.24% of the Italian PTSD patients, by 42% of a group of refugees psychiatric patients in Asian populations [20], by 5.35% in Taiwanese general population (both for hypertension) [21], or by 15% in Australian Vietnam war veterans [26]. An explanation why the stress of the trauma is associated with diseases of the circulatory system may be related to an overactive sympathetic nervous system, haemodynamic reactivity, impaired sympathetic and cardiovagal baroreflex sensitivity, and increased inflammation that could contribute to cardiovascular risk [27].

Lastly, with regard to cancer, it was detected in 8.33% (a generic neoplasm) of Italian PTSD patients but was reported by 42.2% (basal cell carcinoma or squamous cell carcinoma) of subjects enrolled in the study of McLeay and colleagues [25]. Again, the comparison between the comorbidities assessed in the Italian PTSD group and the prevalence among the general population, showed the same problem of data comparability, due to the lack of health information coded through the ICD9CM categories. In this study, 17.86% of the PTSD patients had a metabolic syndrome diagnosis, a percentage which was near to the estimation provided by Miccoli et al. for the Italian population [28]. However, in our study, this disease was more common in women than in men (18% vs. 15%), the opposite of the findings concerning the Italian group of PTSD patients, where the proportion of subjects with metabolic syndrome was 5.56% for women and 27.08% for men. Looking at the ICD9CM code related to neoplasm, this study showed a quite high percentage of subjects affected by this disease (8.33%), with a significant gender difference, since 16.67% of females were diagnosed for neoplasms, compared to 2.08% of males. Data concerning Italian general population did not confirm this difference: according to the AIRTUM Working Group [29], the prevalence in Italy during 2014 was 4.56%, with no substantial difference between females (4.90%) and males (4.20%). The quite high prevalence of neoplasms in PTSD patients may be attributed to a number of immune changes including increased circulating inflammatory markers, increased reactivity to antigen skin tests, lower natural killer cell activity, and lower total T lymphocyte counts [30].

Much more difficult was the comparison with the Italian general population for the diseases of the circulatory system. Giampaoli et al. [31] estimated that in Italy during the period 2008-2012, 1.6% of men and 0.6% of women suffered from acute myocardial infarction, 52.5% of men and 37.8% of women were diagnosed for hypertension and 0.7% (both sexes) experienced cerebrovascular accidents. In this study, a percentage of 20.24% PTSD patients reported a disease of the circulatory system, with a significant gender difference (29.16% males vs. 8.33% females) that is confirmed by the literature and by the general population health status.

In conclusion, this study conducted on a group of Italian PTSD patients confirmed the literature findings on the association between this disorder and medical conditions. Overall, comorbidities showed a high frequency and a higher prevalence than in the Italian general population. Results suggested also that gender may play an important role in developing comorbidities when subjects are affected by PTSD, as suggested by other studies [8]. Our study suggests that assessment and treatment of PTSD patients during clinical and forensic practice should take into account such comorbidities. Due to the limitations of the study design and the difficulty comparing these findings with national and international data, this research brings out the need for deepening this topic in the Italian population.

## Limitations

Although the group of subjects was recruited in a hospital setting and in a national observatory specifically dedicated to PTSD patients, it cannot be considered a true sample of Italian PTSD subjects. The study design did not allow a robust prevalence estimation for each comorbidity since it was not a true cross-sectional study. Another limitation concerns the lack of a comparison group with patients having other psychiatric disorders (e.g., anxiety/depressive disorders) which can be associated with physical comorbidities [32, 33].

## Data Availability

All data generated or analyzed during this study are included in this published article.

## Abbreviations

CAPS: Clinician-Administered PTSD Scale
DTS: Davidson Trauma Scale
ICD: International Classification of Diseases
ICD9CM: International Classification of Diseases, Ninth Revision, Clinical Modification
MINI: Mini International Neuropsychiatric Interview
PTSD: post-traumatic stress disorder
